# Criterion and Construct Validity of the CogState Schizophrenia Battery in Japanese Patients with Schizophrenia

**DOI:** 10.1371/journal.pone.0020469

**Published:** 2011-05-26

**Authors:** Taisuke Yoshida, Motomu Suga, Kunimasa Arima, Yasuko Muranaka, Tsunehiko Tanaka, Satoshi Eguchi, Crystal Lin, Sumiko Yoshida, Masanori Ishikawa, Yuko Higuchi, Tomonori Seo, Yoshinori Ueoka, Masahito Tomotake, Yasuhiro Kaneda, David Darby, Paul Maruff, Masaomi Iyo, Kiyoto Kasai, Teruhiko Higuchi, Tomiki Sumiyoshi, Tetsuro Ohmori, Kiyohisa Takahashi, Kenji Hashimoto

**Affiliations:** 1 Division of Clinical Neuroscience, Chiba University Center for Forensic Mental Health, Chiba, Japan; 2 Department of Psychiatry, Chiba University Graduate School of Medicine, Chiba, Japan; 3 Department of Neuropsychiatry, Graduate School of Medicine, University of Tokyo, Tokyo, Japan; 4 Department of Psychiatry, National Center Hospital, National Center of Neurology and Psychiatry, Tokyo, Japan; 5 National Center of Neurology and Psychiatry, Tokyo, Japan; 6 Department of Neuropsychiatry, University of Toyama Graduate School of Medicine and Pharmaceutical Sciences, Toyama, Japan; 7 Department of Psychiatry, Course of Integrated Brain Sciences, Medical Informatics, Institute of Health Biosciences, University of Tokushima Graduate School, Tokushima, Japan; 8 Department of Psychiatry, Iwaki Clinic, Tokushima, Japan; 9 CogState Ltd., Melbourne, Australia; 10 Japan Foundation for Neuroscience and Mental Health, Tokyo, Japan; Rikagaku Kenkyūsho Brain Science Institute, Japan

## Abstract

**Background:**

The CogState Schizophrenia Battery (CSB), a computerized cognitive battery, covers all the same cognitive domains as the Measurement and Treatment Research to Improve Cognition in Schizophrenia (MATRICS) Consensus Cognitive Battery but is briefer to conduct. The aim of the present study was to evaluate the criterion and construct validity of the Japanese language version of the CSB (CSB-J) in Japanese patients with schizophrenia.

**Methodology/Principal Findings:**

Forty Japanese patients with schizophrenia and 40 Japanese healthy controls with matching age, gender, and premorbid intelligence quotient were enrolled. The CSB-J and the Brief Assessment of Cognition in Schizophrenia, Japanese-language version (BACS-J) were performed once. The structure of the CSB-J was also evaluated by a factor analysis. Similar to the BACS-J, the CSB-J was sensitive to cognitive impairment in Japanese patients with schizophrenia. Furthermore, there was a significant positive correlation between the CSB-J composite score and the BACS-J composite score. A factor analysis showed a three-factor model consisting of memory, speed, and social cognition factors.

**Conclusions/Significance:**

This study suggests that the CSB-J is a useful and rapid automatically administered computerized battery for assessing broad cognitive domains in Japanese patients with schizophrenia.

## Introduction

Cognitive impairment, a core symptom of schizophrenia, is present at illness onset and usually persists even when psychotic symptoms have been successfully treated [1], [2]. Furthermore, cognitive impairment is highly related to functional outcome in patients with schizophrenia [3], [4]. Therefore, treatment of cognitive impairment is currently an important focus for psychopharmacology [5]–[10].

In contrast, the lack of an accepted standard battery for measuring cognitive impairment in patients with schizophrenia had been a major obstacle to regulatory approval of cognition-enhancing treatments. Currently, National Institute of Mental Health - Measurement and Treatment Research to Improve Cognition in Schizophrenia (MATRICS) initiative - Consensus Cognitive Battery (MCCB) is available for the measurement of cognitive changes in patients with schizophrenia [11], [12]. The MCCB has seven domains of cognitive function, including verbal learning, speed of processing, attention/vigilance, working memory, visual learning, reasoning and problem solving, and social cognition [11]. The MCCB was approved by Food and Drug Administration for use in clinical trials for cognitive improvement in schizophrenia [13]. However, a Japanese version of the MCCB is not yet available. In contrast, the Japanese language version of the Brief Assessment of Cognition in Schizophrenia (BACS) [14], [15] has been used to measure cognitive impairments in Japanese patients with schizophrenia.

Like the BACS, the CogState Schizophrenia Battery (CSB) has been developed to provide a briefer standardized assessment of cognition in schizophrenia. Although the BACS includes only four of the seven cognitive domains of the MATRICS initiative [16], [17], the CSB includes all the seven cognitive domains [18], [19]. Formal validation studies have shown the CSB to have very good sensitivity to cognitive impairment in patients with chronic schizophrenia, and require approximately 40 min for administration [18], [19]. There is also a strong correlation between the composite scores from the CSB and the MCCB measures in patients with schizophrenia. Furthermore, both composite scores also correlate strongly with scores on Performance-Based Skills Assessment [19]. Importantly, because the CSB was developed specifically for the measurement of cognitive change the component tasks show minimal practice effects with repeated assessment, even during very brief re-test intervals [19].

The aim of the current study is to assess the validity of the Japanese language version of the CSB (CSB-J) in Japanese patients with schizophrenia by comparing performance on this battery to that of the Japanese language version of the BACS (BACS-J) already validated for use in Japan.

## Methods

### Subjects

Forty patients with schizophrenia were recruited at Chiba University Hospital (Chiba, Japan), The University of Tokyo Hospital (Tokyo, Japan), National Center Hospital, National Center of Neurology and Psychiatry (Tokyo, Japan), Toyama University Hospital (Toyama, Japan), and Tokushima University Hospital (Tokushima, Japan). All patients met the DSM-IV criteria for schizophrenia. No patient had received electroconvulsive therapy. There were no specific medication criteria for inclusion in the patient group. Twenty-five of 40 patients were treated with a single second-generation antipsychotic medication (risperidone, n = 8; aripiprazole, n = 7; olanzapine, n = 6; perospirone, n = 3; quetiapine, n = 1), four patients were treated with a single first-generation antipsychotic (haloperidol, n = 1; fluphenazine, n = 1; bromperidol, n = 1; sulpiride, n = 1), nine patients were treated with a combination of antipsychotic drugs (aripiprazole and quetiapine, n = 2; risperidone and quetiapine, n = 1; risperidone and haloperidol, n = 1; risperidone and levomepromazine, n = 1; haloperidol and levomepromazine, n = 1; haloperidol and zotepine, n = 1; risperidone, haloperidol, and bromperidol, n = 1; risperidone, haloperidol, and zotepine, n = 1), and two patients were medication free. Only two female patients were inpatients.

Forty healthy controls were recruited at the same five sites. They were screened with the Structured Clinical Interview for DSM-IV Axis I Disorders, Non-Patient Edition and were required not to have an Axis I disorder according to DSM-IV criteria. None had a first-degree family history of schizophrenia or schizoaffective disorder.

Inclusion criteria for all subjects in both groups included proficiency in Japanese language, normal or corrected-to-normal visual function, and at least a 9th-grade education. Exclusion criteria for all subjects in both groups included any current or past histories of neurological disorders (other than schizophrenia for the patient group), including head injury, cerebral vascular disorders, epilepsy, or alcohol or drug use disorders. No subject was treated with donepezil. Participants who had severe symptoms of depression (defined by the Japanese version of the Calgary Depression Scale for Schizophrenia (JCDSS) [20], [21] score of more than 9) were excluded from the study. Smokers were excluded because nicotine and nicotine withdrawal might have effects on cognition.

Study investigators made a concerted effort to recruit healthy controls who would match the patients on age, male/female ratio, and premorbid intelligence quotient (IQ) as assessed by the Japanese Adult Reading Scale-25 words version (JART-25) [22], which is Japanese version of National Adult Reading Test. Age was considered the primary demographic variable of interest since it was likely to have the greatest impact on cognition. The 40 subjects of both groups were divided into 4 age groups (1, 20–29 years old; 2, 30–39 years old; 3, 40–49 years old; 4, 50–65 years old). Prior to commencement of the study, all subjects provided written informed consent after receiving a full explanation regarding the nature of the study and potential risks and benefits of study participation. The study was approved by the relevant ethics committee of each institute and performed in accordance with the Declaration of Helsinki II. The ethics committees of each institute were: the Ethics Committee of Chiba University Graduate School of Medicine (Chiba, Japan), the Ethical Committee of the Faculty of Medicine, University of Tokyo (Tokyo, Japan), the Ethics Committee of National Center of Neurology and Psychiatry (Tokyo, Japan), the Committee on Medical Ethics of Toyama Medical and Pharmaceutical University (Toyama, Japan), and the Ethics Committee of University of Tokushima (Tokushima, Japan).

### Assessment procedures

All subjects completed two batteries of cognitive tests administered by trained psychiatrists or psychologists. All subjects received the CSB-J followed by the BACS-J version A. JART-25 was completed after the BACS-J. All subjects were tested in a single day. In addition, the Positive and Negative Syndrome Scale (PANSS) [23] was completed along with the BACS-J. Short breaks of five minutes or less were provided as needed throughout testing. Subjects were instructed to avoid caffeine in all forms from 20 minutes prior to assessments to the end of all tests.

The CSB-J consists of eight tasks that measure verbal learning (International Shopping List Task; ISLT), speed of processing (Detection Task; DET), attention/vigilance (Identification Task; IDN), visual working memory (One Back Task; ONB), visual memory (One Card Learning Task; OCL), spatial working memory (Continuous Paired Association Learning Task; CPAL), reasoning and problem solving (Groton Maze Learning Task; GML), and social cognition (Social Emotional Cognition Task; SECT). The primary measure from each task of the CSB-J was standardized by creating Z-scores whereby healthy control mean was set to zero and the standard deviation set to one, following the methodological procedure used by Keefe et al. [14]. A composite score was calculated by averaging all Z-scores of the eight primary measures from the CSB-J. In this study, we used the original version of the CSB with a slight modification. First, the Two Back Task was omitted to reduce test duration because we considered the ONB sufficient to assess working memory function [19]. Second, the CPAL can provide another non-verbal paired associate learning [24]. Third, the list of words in the ISLT was customized for the study as recommended by the authors to match regional Japanese culture and minimize cross-cultural test bias [25]. Fourth, stimuli in the SECT were also customized to only include faces with a Mongoloid countenance to avoid any other-race effects that can occur on tasks that use representations of human faces [26].

The CSB-J data were uploaded to a secure account on the CogState server (http://www.cogstate.com). Uploaded outcome parameters were calculated using custom software blind to diagnosis. Logarithmic and arcsine transformations for speed and accuracy measures respectively were performed in order to avoid violation of necessary statistical preconditions. A description of the battery's administration and the eight cognitive tasks has been reported previously for non-Japanese subjects [19], [27].

### Data analysis

Student's t-test and Fisher's exact test were used to examine differences between groups. For the analysis of concurrent validity, Pearson product-moment correlations were computed between scores on subtests of the CSB-J and the BACS-J within each cognitive domain. Stepwise General Linear Models (GLM) with the CSB-J composite score or subscores as the dependent variable were conducted. At first, with combined patients' and controls' data, GLM were used to evaluate the effects of the following independent variables on cognitive performance: age, sex, premorbid IQ, education, JCDSS score. Second, with patients' data, GLM were used to evaluate the effects of the following independent variables on cognitive performance: age, gender, premorbid IQ, illness duration, duration of untreated psychosis (DUP), the dosage of antipsychotic medication, the dosage of anticholinergic medication, PANSS positive syndrome scale score, PANSS negative syndrome scale score, PANSS general psychopathology scale score. The structure of the CSB-J was determined by performing the Maximum Likelihood extraction methods with oblique rotation. The Kruskal-Wallis test was used to compare cognitive impairment among different subtypes of schizophrenia. Values of *p*<0.05 were considered to indicate statistical significance.

## Results

### Demographic data and clinical variables

Demographic and clinical variables are presented in Table 1. Age, gender, estimated premorbid IQ and education did not differ for the two groups. The JCDSS score in patients was significantly higher than that of healthy controls, indicating that the schizophrenia patients group suffered more depressive symptoms.

**Table 1:** Demographic and symptom information.

	Controls (n = 40)	Patients (n = 40)	*p*-value
Age (years)	39.6±11.9 (22–59)	39.6±12.3 (22–65)	1.000
Male/Female	20/20	20/20	1.000
Premorbid IQ	107.1±8.5 (89–120)	103.7±10.1 (79–120)	0.114
Education (years)	15.0±1.8 (12–20)	14.3±2.0 (10–20)	0.139
JCDSS	0.6±1.4 (0–6)	2.2±2.4 (0–9)	0.001
Illness duration (years)		15.6±11.6 (2–38)	
Duration of untreated psychosis (years)		2.5±6.0 (0–37)	
Chlorpromazine equivalents (mg)		410.8±305.6 (0–1250)	
Biperiden equivalents (mg)		1.0±1.9 (0–6)	
PANSS positive		14.1±5.2 (6–24)	
PANSS negative		17.9±6.1 (9–36)	
PANSS general		33.1±10.9 (18–47)	

Data are the mean ± S.D. Parenthesis is the range.

JCDSS: the Japanese version of Calgary Depression Scale for Schizophrenia.

PANSS: Positive and Negative Syndrome Scale.

### Missing data across all sessions and administration time

The total amount of missing data across all tasks within the CSB-J was 1.25%. The reason for missing data was the time restriction of each subtest of the CSB-J. There was no missing data for the BACS-J subtests. The total administration time of the CSB-J (51.1±12.2 min (mean ± SD)) was significantly (t = 10.719, *p*<0.001) longer than that of the BACS-J (35.6±4.4 min (mean ± SD)).

### Validity and stepwise analysis

**Figure 1:**
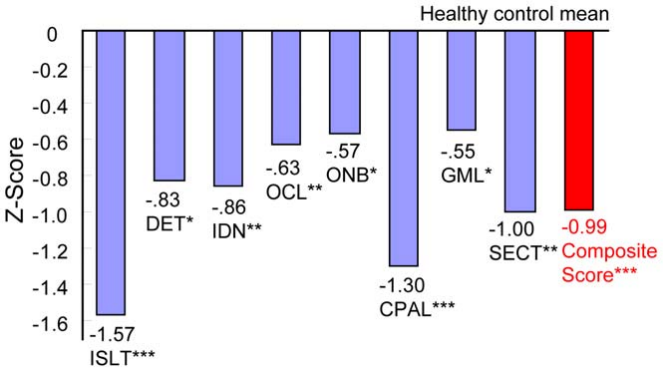
Magnitude of impairment relative to matched healthy controls on each cognitive measure from the CSB-J. Abbreviation: ISLT International Shopping List Task, DET Detection Task, IDN Identification Task, OCL One Card Learning Task, ONB One Back Task, CPAL Continuous Paired Association Task, GML Groton Maze Learning Task. Numbers of the figure are Z-score. *p<0.05, **p<0.01, ***p<0.001.

**Figure 2:**
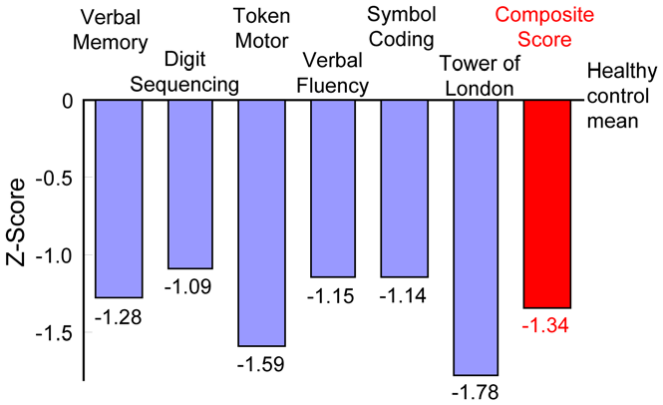
Magnitude of impairment relative to matched healthy controls on each cognitive measure from the BACS-J. Numbers of the figure are Z-score. All subtests and composite score were *p*<0.001.

Figure 1 and 2 shows the performance of patients on each of the primary measures and composite score of the CSB-J and the BACS-J compared to the healthy control, respectively. Significant differences in scores between the patients and the controls were observed for all of the subtests of the CSB-J and the BACS-J.

**Figure 3:**
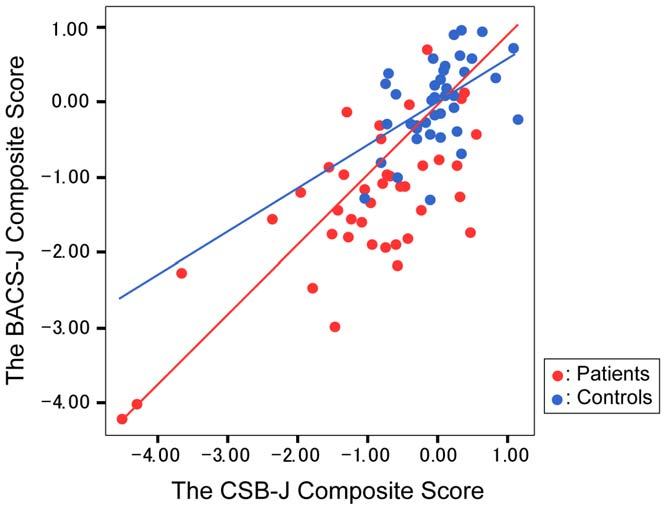
Inter-correlations between the CSB-J composite score and the BACS-J composite score. Controls: r = 0.483; p<0.01, Patients: r = 0.709; p<0.001, Total subjects: r = 0.760; p<0.001.

The CSB-J composite score was significantly correlated with the BACS-J composite score (*r* = 0.709; *p*<0.001 for patients, *r* = 0.483; *p*<0.01 for controls; *r* = 0.760; *p*<0.001 for total subjects) as shown in Table 2 and Figure 3. Stepwise GLM showed that age and premorbid IQ were independent predictors of the CSB-J composite scores. Lower cognitive performance was associated with increased age and lower premorbid IQ. After accounting for age and premorbid IQ, the difference between both composite scores remained. Other clinical variables were not correlated with the CSB-J composite score.

**Table 2:** Correlation efficiencies between the CSB-J and the BACS-J for the same cognitive domains.

Cognitive domain		Patients	Controls	Totals
Verbal learning	International Shopping List Task vs. BACS-J Verbal memory	.725***	.424**	.714***
Speed of processing	Detection Task vs. BACS-J Token motor	.105	.025	.207
	Detection Task vs. BACS-J Verbal fluency	−.184	−.031	−.003
	Detection Task vs. BACS-J Symbol coding	.466**	−.167	.341**
Working memory	One back Task vs. BACS-J Digit sequencing	.169	−.041	.181
	Continuous Paired Association Task vs. BACS-J Digit sequencing	.192	.284	.342**
Reasoning and problem solving	Groton Maze Learning Task vs. BACS-J Tower of London	.135	.276	.250*
Composite Score	The CSB-J Composite Score vs. The BACS-J Composite Score	.709***	.483**	.760***

*p<0.05,

**p<0.01,

***p<0.001.

Next, we examined correlations between corresponding subtests from the CSB-J and the BACS-J. Because the BACS-J includes only four of the seven cognitive domains selected by the MATRICS initiative, we examined correlations of corresponding subtests in only these four domains. ISLT score and DET score were significantly correlated with the BACS-J verbal memory score (*r* = 0.725, *p*<0.001) and the BACS-J symbol coding score (*r* = 0.466, *p*<0.01) in patients, respectively. There were no significant correlations between other corresponding subtests (Table 2).

**Figure 4:**
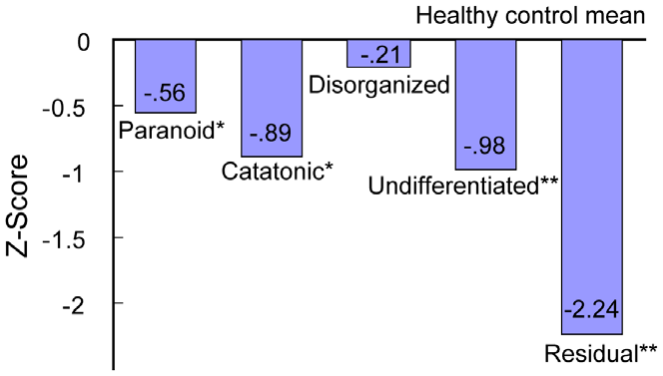
Effects of subtype on CSB-J composite score in patients with schizophrenia. Number of the figure is Z-score. *P<0.05, **P<0.01 as compared with control.

Furthermore, we examined the effect of five subtypes of schizophrenia on the CSB-J scores in patients with schizophrenia although the number of each subtype was small. The CSB-J score in each subtype is shown in Table S1. The disorganized subtype (n = 3) demonstrated intact cognition. The paranoid subtype (n = 20) and the catatonic subtype (n = 4) performed significantly worse on ISLT and the CSB-J composite score than controls. The undifferentiated subtype (n = 4) performed significantly worse on ISLT, IDN, and composite score than controls. The residual subtype (n = 9) performed significantly worse on broader domains than controls, and had stronger impairment on the CSB-J composite score (Figure 4 and Table S1).

### Factor analysis of the CSB-J subtests

In a factor analysis of the CSB-J, the eigenvalue-greater-than-one rule and scree plot converged on a three-factor solution that accounted for 53.8% of the total variance. The Kaiser-Mayer-Olkin measure was calculated at 0.587 and Bartlett's test of sphericity was significant at *p*<0.001. The factor loadings are presented in Table 3. Subtests that needed memory loaded on Factor 1, including CPAL, OCL, ISLT, and GML. Subtests that needed speed loaded on Factor 2, including DET and IDN. The SECT loaded on Factor 3. The ONB was not associated with this three-factor solution.

**Table 3:** Factor loading of the CSB-J subtests in patients with schizophrenia.

	Factor 1	Factor 2	Factor 3
Continuous Paired Association Learning Task	**.** ***912***	.028	.063
One Card Learning Task	**.** ***552***	.006	−.211
International Shopping List Task	**.** ***517***	−.127	−.177
Gorton Maze Learning Task	**.** ***432***	.053	.372
Detection Task	.116	**.** ***987***	−.136
Identification Task	−.202	**.** ***734***	.224
Social Emotional Cognition Task	−.025	−.037	**.** ***908***
One Back Task	−.174	.077	.365

## Discussion

The present study is the first one to report the use of a complete MCCB compatible battery in Japanese schizophrenia patients and shows that the CSB-J is a useful neuropsychological battery for assessing global cognitive impairment in Japanese patients with schizophrenia. The CSB-J was easy to use and well tolerated by patients with a 98.8% completion rate and acceptable administration time with mean of 51.1 minutes. Although the administration time of the CSB-J was about 15 minutes longer than that of the partial MCCB BACS-J battery (with average of 35.6 minutes administration duration), the difference was probably in part because the CSB-J covered more cognitive domains than the BACS-J. In addition, there was a significant correlation between the CSB-J and the BACS-J composite scores in both the patients with schizophrenia and healthy control subjects groups, consistent with the previous results using the original English version of the CSB and standardized tests and the MCCB [18], [19].

The results of this study also provide evidence of good construct validity for verbal memory and attentional domains between the CSB-J and BACS-J tasks, which are considered to evaluate these abilities. In particular, the ISLT and DET scores of the CSB-J were significantly correlated with the verbal memory and the symbol coding scores of the BACS-J in patients, respectively. However, there were no significant correlations between the other subscores of the CSB-J and the corresponding subscores of the BACS-J that are considered to evaluate speed of processing, working memory, and reasoning and problem solving. These differences presumably relate to different task requirements. For example, the BACS-J token motor test requires an ability to coordinate both hands simultaneously, whilst the CSB-J IDN task requires simpler motor abilities for pushing the response buttons. Prior good correlations for the IDN task and information processing speed measures have been reported in non-Japanese schizophrenic patients [18], and a poor correlation between the token motor test and a corresponding conventional test [14], [28], suggest that these tests measure differing abilities. Likewise, verbal fluency is associated with multiple cognitive abilities, including speed of processing, reasoning ability and other aspects of executive function such as inhibition [29]. Similarly, the tasks evaluating working memory from the different batteries had significant differences. The ONB and CPAL tasks using the CSB-J probably correlate with visual and spatial working memory, whilst the digit sequencing of the BACS-J may correlate less with visual and more with verbal working memory. With respect to reasoning and problem solving, although both the GML task of the CSB-J and the Tower of London from the BACS-J require planning, inhibition, and working memory, the latter has been considered more of a planning task [30], whereas the GML task appears to highlight spatial working memory abilities [31]. Differences between the constructs evaluated by these two batteries appear a more salient explanation for the lack of correlations, since both the CSB-J subtests and the BACS-J subtests have been reported to be significantly correlated with the corresponding standard battery subscores [15], [19].

The factor analysis performed on the CSB-J suggests that three factors of cognitive performance can be derived from the CSB-J scores. The first factor had memory as a common ability and included the CPAL, OCL, ISLT, and GML tasks. A second speed of performance factor included the DET and IDN tasks, and a third factor separated out the SECT task, which includes abilities collectively considered important in social cognition. It has been suggested that social cognition represents a separate cognitive domain in schizophrenia [32]. Social cognitive ability is considered to be an important predictor of effective social [33] and community function (including interpersonal relationships and work functioning) independent of abilities in other cognitive domains [34], [35]; however we did not perform additional social and community functional assessments in this study. Taken together, the CSB-J may have an advantage over the BACS-J because the BACS-J lacks a social cognition subtest.

Although the numbers of each diagnostic subtype of schizophrenia were small in this study, we did find that each subtype had a quite different profile of CSB-J score. Both the undifferentiated subtype and the residual subtype had major cognitive impairment on the CSB-J composite score, consistent with previous reports [36], [37]. In contrast, Brazo et al. [38] reported that the disorganized subtype had major cognitive impairments, whereas in our study the disorganized subtype had intact cognitive function. The reasons underlying this discrepancy are currently unknown. Clearly a larger sample will be required to further investigate this issue.

There are some limitations of this study. First, some subtests of the CSB-J were not assessed in the criterion-related validity analysis. This is because of the absence of equivalent MCCB domain specific tests for Japanese. Second, the assessment of social cognition by emotional perception alone does not cover many of the putative abilities thought to underlie this complex behavior. Further studies will be required if other social and emotional cognitive tasks are adapted for Japanese patients. Third, the sample size of this study was small (n = 40 for each group), and larger studies would aid in confirming and extending the findings of the current study. Further detailed studies of the CSB-J in comparison to a complete Japanese language version of the MCCB and other social cognitive abilities such as theory of mind and attributional style would help determine the applicability of this promising battery. Furthermore, the current study did not repeat the batteries precluding assessment of test-retest validity, which is considered by the MATRICS initiative investigators a vital feature of a test battery to be used in clinical trials of schizophrenia [11]. Since test-retest results have been reported for both the CSB and the MCCB in non-Japanese control subjects and schizophrenic patients [39]–[41], such studies using Japanese samples are recommended.

In conclusion, the present study showed that the CSB-J was sensitive to cognitive impairment in Japanese patients with schizophrenia, and that the CSB-J composite score was significantly correlated with the BACS-J composite score providing initial criterion and construct validation. Although further studies are required to address test-retest validity, the CSB-J appears to be a promising cognitive battery to assess the therapeutic effects on potential cognitive-enhancing agents in Japanese patients with schizophrenia.

## Supporting Information

Table S1 **The CSB-J subscores of each subtype of schizophrenia.** *p<0.05, **p<0.01 (for post-hoc analysis). Kruskal-Wallis tests; post-hoc tests; comparison between each subtype and controls. The comparison procedure was appropriately adjusted by reducing the level of significance (Bonferroni procedure). ISLT: International Shopping List Task, DET: Detection Task, IDN: Identification Task, ONB: One Card Learning Task, CPAL: Continuous Paired Association Learning Task, GML; Gorton Maze Learning Task, SECT: Social Emotional Cognition Task.
(DOCX)
