# Supplementary material for: Criterion and Construct Validity of the CogState Schizophrenia Battery in Japanese Patients with Schizophrenia
Source: PLoS One. 2011 May 26;6(5):e20469. doi: 10.1371/journal.pone.0020469 (PMC3102733; doi:10.1371/journal.pone.0020469)
Supplement: Table S1 — The CSB-J subscores of each subtype of schizophrenia. *p<0.05, **p<0.01 (for post-hoc analysis). Kruskal-Wallis tests; post-hoc tests; comparison between each subtype and controls. The comparison procedure was appropriately adjusted by reducing the level of significance (Bonferroni procedure). ISLT: International Shopping List Task, DET: Detection Task, IDN: Identification Task, ONB: One Card Learning Task, CPAL: Continuous Paired Association Learning Task, GML; Gorton Maze Learning Task, SECT: Social Emotional Cognition Task. (DOCX) [file pone.0020469.s001.docx]

**Table S1. The CSB-J subscores of each subtype of schizophrenia.**

| **CSB-J score** | **Controls (n=40)** | **Paranoid (n=20)** | **Catatonic (n=4)** | **Disorganized (n=3)** | **Undifferentiated (n=4)** | **Residual**  **(n=9)** | ***P*** |
| --- | --- | --- | --- | --- | --- | --- | --- |
| ISLT | 0±1 | -1.11±1.37** | -1.95(1.15)* | -.60±1.59 | -1.53±.88* | -2.75±.95** | <.001 |
| DET | 0±1 | -.34±1.05 | -.39±.92 | 0.18±1.57 | -1.13±2.76 | -2.31±3.55 | .352 |
| IDN | 0±1 | -.54±1.01 | -.12±1.11 | -.33±.48 | -.1.36±1.07* | -2,08±2.41 | .033 |
| OCL | 0±1 | -.49.±.89 | -.53±.86 | .28±0.54 | -.01±.63 | -1.54±.88** | .003. |
| ONB | 0±1 | -.33±1.38 | -.66±.51 | -.80±2.15 | -1.17±1.18 | -.74±1.66 | .253 |
| CPAL | 0±1 | -.64±1.19 | -1.47±.96 | -1.02±1.24 | -.73±1.31 | -3.27±3.80** | <.001 |
| GML | 0±1 | -.32±1.35 | -.83±1.59 | -.13±.04 | -.69±1.02 | -1.41±1.83 | .278 |
| SECT | 0±1 | -.68±1.51 | -1.16±2.59 | 0.08±1.90 | -1.18±1.35 | -2.16±3.07 | .157 |
| Composite Score | 0±1 | -.56±.72* | -.89±.78* | -.21±.55 | -.98±.36** | -2.24±1.55** | <.001 |
